# Messrs. Dalbant, Vaidy, and Others, on Arterial Inflammation

**Published:** 1821-06-01

**Authors:** 


### ARTERIAL INFLAMMATION.

### IV.

1. *Quelques Observations pour servir à l'Histoire de l'Arterite, ou Inflammation des Artères.* Par M. Dalbant, a Paris. Juillet, 1819.
2. M. Vaidy *in Journal Complimentaire,* Août 1819.
3. M. Vaidy *in Revue Medicale.* Mai, 1820.

Inflammation is an abstract term employed in medical philosophy to express what is generated in a living part by the influence of certain causes whose particular effects determine inordinate action in the nervous and vascular organs of that part. This what is not an *entity,* a *thing,* because it does not exist as an essential substance: it is a *condition, a state,* because it consists in the external qualities of the living part which is its seat; and it is a *morbid state,* because it depends on the presence of new and unnatural or unhealthy accidental differences introduced into the external qualities of the part, by whatever agent may be their determinative cause. Inflammation, then, is a morbid state, a disease, dependent on the circumstances of organic matter; and, consequently, is not peculiar to the human race. Animals, throughout all their tribes, are exposed to suffer from any of the modifications which its varieties may assume. Being thus uninfluenced by the conditions of man's intellectual nature, its manifestations appear only in the derangements of his vital actions, and his organic structure alone can become the subject of those phenomena which attend the development of inflammatory disease.

Vascular inflammation is produced by those causes which determine irregular action in the nerves and *vasa vasorum,* or vessels whose function is to supply and maintain the structural elements of the lymphatic and sanguiferous organs. Our present researches, however, shall be restricted to an investigation of the distinctive forms, and their pathology, as existing in a morbid state of the *arterial* tubes.

Arteries, being composed of the elementary principles peculiar to all the diversities of animal structure, are consequently exposed to the same morbid changes in their vital actions. Inflammation of these vessels, previously to the time of those authors, from whose writings, and the records of our own experience, we propose to select the materials of this article, had been as unfrequently noted as it was imperfectly described. We shall, therefore, be somewhat particular in our endeavours to illustrate its causes, nature, and influences; hoping that these our labours may, in some degree, contribute to the better understanding of an untractable disease.

I. Etiology. Arteritis may be modified, in its nature and degree, by a variety of causes which predispose the arterial textures to become the seat of inflammatory action. Among the *predisponent causes* may be enumerated—the plethoric idiosyncracy—indulgence in the use of rich foods and drinks—influences of climate—effects of inflammatory and febrile affections—suppression of established periodical or permanent discharges, especially the sanguineous—reiterated paroxysms of convulsive, spasmodic, and other nervous diseases—unrestrained sway of the irascible passions—intemperate and luxurious habits of all kinds, particularly such as possess a tendency to induce or confirm an excess of action in the chylopoietic and sanguiferous vessels.

Arterial inflammation is often complicated with other organic diseases; and, not unfrequently, it arises from causes whose progressive operation cannot be retraced. It may be determined by compression, insolation, congelation—by punctured, incised, contused, and lacerated wounds, originating from chirurgical or traumatic agency—by vital exhaustion, mental agitation, violent and forcible exertion, atmospherical vicissitudes—by the mechanical or chemical effects of extraneous substances, solid, fluid, or gazeous—by contact of other morbid organs—and by many of the exciting causes from which particular inflammations proceed.

II. Symptomatology. Arterial inflammation often completes an insidious and fatal course without its existence being ever indicated by the presence of one distinctive sign. The traces of its ravages, in such instances, remain for ever unknown, or, by the researches of pathological anatomy, are incidentally detailed and explained. Our own limited observation inclines us to conclude, that its origin and formation are many times obscured by the indefinite nature of its symptomatic effects. Sometimes, especially when its determining causes escape notice, its symptoms are indistinguishable from those by which the fever, called *synochal*, is accompanied: in other cases, they differ little from those connected with sub-acute or chronic inflammation of the larger abdominal and thoracic organs. When the disease, however, resigns the latent, and assumes a manifest, character, its progress is not different, and its symptoms vary not, from those which mark it when consecutive to an ascertainable or violent cause.

Early in the disease, if its development is not sudden, the patient is restless, impatient, watchful, irritable: he experiences partial flushings, which gradually increase both in frequency and extent: his bowels are inactive, but his pulse is imperceptibly affected. By and bye, he complains of a deep-seated pain in some part of the abdominal or thoracic regions, and seldom fails to describe it as being hot, lancinating, spasmodic, and increased by slight exertion. Disturbance of the vascular system now supervenes: respiration is accelerated, and the breath feels offensive from its heat. The mouth, however, remains humid, the tongue red, the lips moist and natural. Rigors at last are felt; and, as the disease advances, the internal pain becomes more diffused. The original flushings give place to a true febrile state of the cutaneous surface, and the disquietude is succeeded by head-ache, nausea, languor, and depression. Inappetency and quenchless thirst prevail. The bowels are more obstinate; the mouth tastes bitter. The pulse, in some instances, is hard, strong, and tumultuous; in others, wiry and irregular. If transient sleep is obtained, it is unrefreshing and interrupted by startings and frightful dreams. About this time cough begins. This, sometimes, is dry, frequent, teazing; sometimes it recurs in violent and tedious paroxysms, and is accompanied with expectoration of fetid mucus, the clots of which are occasionally streaked with florid blood. When the disease has, at length, acquired a formidable ascendency, all its previous symptoms, and the symptomatic fever, (which is generally the synochal or inflammatory) are greatly aggravated. The patient's countenance suddenly shrinks, becomes pallid, haggard, cadaverous. His strength sinks; his emaciation is extreme. His lips are crimson or livid; his tongue red, smooth, moist. At the same time his breathing is rapid, difficult, irregular; the head-ache intense; the pulse hard, labouring, intermittent, accelerated to 110—130. Consecutive to the former deep, internal pain, a distressful sense of constriction, most frequently in the cardiac, præcordial, or epigastric region, is established. Spasms not unlike those of tetanus, in many cases, occur under the xyphoid cartilage, and in the line of the diaphragm. The countenance and extremities grow œdematous. Forcible palpitations of the heart, and, in some instances, of the abdominal aorta, so as to be distinctly felt through the integuments, now predominate. The arteries pulsate with alarming and painful violence, and the action of the more superficial is quite visible: that of the carotids is audible by the patient himself, often by his attendants. Syncopé is frequent; the paroxysms of dyspnœa protracted and agonizing. The anasarcous effusion extends and becomes general. In many cases ascites supervenes; in others, hydrothorax; in some, the extravasation of serum is universal and profuse. Orthopnœa succeeds, and the patient is confined to a sitting posture. The least motion excites cough, and a sense of instant suffocation. Wheals and ecchymosed patches arise in different parts of his body. Violent convulsions ensue; and, in one of these, exhausted already and moribund, he struggles and expires.

Such, we believe, is not an unfaithful representation of the stages through which the inflammation of arteries pursues its fatal course. Which of all these symptoms, then, shall we regard as distinguishing it from the other diseases to which, by general similarity of character, it may seem to be allied? Until numerous and diversified observations in clinical practice and anatomical pathology shall have farther enlarged our knowledge of the malady, many circumstances will continue to embarrass any attempt towards delineating the symptoms which specificate the arterial from the other acute inflammations which it more intimately resembles. Without going into minute ratiocination on the subject, therefore, we would beg to submit the following practical aphorisms:—1st. That, when the leading symptoms we have just now enumerated do appear, they indicate the existence of inflammatory disease: 2d. That, when the heat and pain, attendant on internal inflammations, cease to be concentrated, this circumstance, connected with the preceding, may suggest the inflammation of some organ whose functions are diffusive, and exercise an extensive influence over the state of the general system: 3d. That, when superinduced to these, the arterial disturbance peculiar to all inflammations is propagated, in an exquisite degree, from the large to the smaller vessels, so as to excite a visible and almost audible impetuosity of action in their superficial ramifications, no error of practice can be imputable to the physician who shall regard the case as one of acute arterial inflammation, and forthwith institute, for its removal, the agency of a vigorous and appropriate treatment.

Arteritis requires, of course, the general treatment adapted to reduce the inflammatory state, in whatever department of the animal economy that state may arise. The peculiar symptoms of the disease may be advantageously combated, we doubt not, by the modified use of such therapeutic agents as are known to contribute to the reduction of inordinate action in the vascular system. More than this, in the present state of our knowledge, we do not deem it wise to advance. We have no theory and few facts to guide us in the investigation, and cannot, therefore, perceive advantage in bestowing, on this branch of the subject, the trouble of an elaborate detail.

III. Pathology. Some idea of the existence and influences of arterial inflammation seems to have been entertained by Aretæus, whose bold and depletory practices are followed and improved by the best physicians of modern times. His words are, speaking of affections of the vena cava—

"Quibusdam, et arteria secundum dorsum inflammatur; quod pulsatio in alteris præcordiis manifestat: nam et ipsa venæ cavæ morborum particeps efficitur juxta illam in sinistro latere posita; ****** somni tumultuosi fiunt; alvus quibusdam nihil reddit, quibusdam exiguum, acre, biliosum, &c." *Lib.* ii. *cap.* viii.

Detached notices of the organic lesions affected by arterial inflammation are also scattered through the writings of subsequent observers; but, being unaccompanied by any description of the pathetic* symptoms, they all are as defective as they are unsatisfactory. The disease and its results, however, did not altogether escape the inquisitive hand of Morgagni, to whose venerable pages the following history has been consigned.

Case.—*Pathology.* "A sexagenarian, who had formerly been syphilitic, was afflicted with rheumatism which, about fifteen years afterwards, yielded to an artificial perspiration. Subsequently, he became corpulent and liable to paroxysms of dry cough, with difficulty of breathing, especially on taking food. In other respects he seemed healthy and robust, but latterly complained of confusion in his head. At last, immediately after taking a spare supper, this man was suddenly seized with a slight cough, which rapidly increased. He became convulsed, and died instantly; emitting a frothy and sanguinolent fluid from his nostrils and mouth.

" *Necrotomy.*† Externally, the sides of the thorax and the countenance were red and livid. Both internally and externally, the lungs were liver coloured and softened. Each of them adhered to the sternal pleura, the left to all the contiguous parts. In each of the thoracic cavities and the pericardium was a large quantity of bloody serum. Both ventricles, as well as auricles of the heart, were empty; its structure sound; its valves and large vessels natural. The caliber of the aorta, from its origin to its arch, was dilated; and over its internal surface, many white spots were interspersed. "*Et, quod mihi præcipuum visum est, colore ex atro rubens*‡ *ut si inflammatione quâ-*

---

* Pathetic. This term has been carried away from the province of medical philology, to which it naturally belongs. By the Greeks, from whose comprehensive language it is derived, the word λογος was employed to denominate a particular disease, while, by παθος, disease in general, on a diseased state, was emphatically expressed. *Pathetic*, in the text, signifies *pertaining to disease, arising from disease, denoting disease;* and the *pathetic symptoms* are those by which we are apprized of *the morbid state*. We submit the propriety of reclaiming this epithet for the use of pathological science.

† *Necrotomy.* We venture to employ this word in the room of *post mortem appearances*, and other incongruous expressions which deform our medical style. It is constructed on principles strictly analogous to those of the English language; and, being derived from Νεκρος, *cadaver ipsum, a corpse, a dead body;* and Τομη, *section, incision, dissection;* is equivalent to the Latin *sectio cadaveris*, and literally signifies *the dissection of a dead body;* which is more appropriate than *autopsia*, which only signifies inspecting, viewing, contemplating by one's self.

‡ Boerhaave discovered in a bullock "qui vehementissimo cursu aufugerat," the aorta "ut esset *nigerrima*." The same distinguished pathologist ranks "inflammatio arteriosa" among the causes of an intermittent pulse. *Institutionum Medicinæ*, Sect. 827.

*dam esset affecta.*" Beyond the arch, at the origin of the innominata, and where the aorta descends near the vertebræ, a few of these spots were also found. The vessels of the pia mater were distended with blood: the lateral ventricles contained a little bloody serum. Every other part of the cerebrum, cerebellum, and medulla oblongata, was perfectly natural. The abdomen was not examined."*

Morgagni, in remarking on the peculiar features of this case, does not deny the possibility of the cough, convulsions, and suffocation, having proceeded from an invisible cause latent in some nervous ganglion, but feels disposed to regard inflammation of the aorta, whether connected or unconnected with the cough and convulsions, as a chief cause of the patient's death.

Schmuck, in 1794, published an essay† on the inflammation of blood-vessels; and, in 1797, was followed in the same path of research by Saase,‡ who superadded to his own all the practical observations of his predecessor, together with a number which Meckel communicated to him, in illustration of his subject. This writer made a variety of experiments, by which he introduced different chemical irritants into the cavities of blood-vessels, and uniformly found the result to be, the formation of false membranes or obliteration of the vessels by clots of coagulable lymph. Subsequently, an analytical translation of the more useful parts of Saase's dissertation was made by Schwilqué, whose memoirs§ also contains a summary of the facts advanced by previous authors, with the design of establishing their particular views of the nature of vascular inflammation. The combined testimony of all these facts is submitted as evidence—that many points of the internal face of veins and arteries may be inflamed—that no person has ever seen all the blood-vessels affected at the same time with inflammation—that veins are more frequently inflamed than arteries —that, whatever be the lesioned vessel, inflammation is *in general* seated in its internal membrane, *sometimes* in the external, and *may extend* itself to the contiguous parts—and that vascular inflammation may terminate in suppuration, in the formation of false membranes, or in thickening of the part in which it acts.

---

* J. P. Morgagni de Sedibus et Causis Morborum, Epist. xxvi. Art. 35. 36. Tom. II. p. 284, 285.

† Dissertatio Sistens Obs. Med. de Vasor. Sanguif. Inflammatione, Heidelbergi, 1794.

‡ Dissertatio de Vasorum Sanguiferorum Inflammatione, Hallæ, 1797.

§ Faite pour servir a l'Histoire des Inflammations Veineuses et Arterielles, inseré dans la Bibliothèque Medicale, Tome xvi.

Inflammation of an artery from the application of a ligature is sometimes propagated along the vessel's internal membrane, to a considerable extent. Œhme* discovered this state of the epigastric artery consequent on ligature of the umbilical cord. After ligature of the femoral artery for aneurism, Mr. Cline† and Mr. Abernethy‡ found traces of inflammation over all the course of that vessel, and of the aorta to its origin in the heart.

Portal, alike venerable for his age and his usefulness, could not in the multitude of his dissections avoid having detected the particular lesions determined by inflammation of the arterial coats. Accordingly, we find him stating,§ as a pathological fact, that blood-vessels are susceptible of inflammatory action, and, at the same time, illustrating this doctrine by the outline of a case which came under his own observation. "Few authors," he says, "have treated of aortal inflammation: I have often discovered traces of it in subjects with the history of whose diseases I was unacquainted; and I am now satisfied of its existence by what I saw in a young man whose body I myself inspected."—This person died of a few days illness, induced by sudden retrocession of measles. His chief symptoms were an exquisite sense of suffocation and violent palpitations of the heart. After death his lungs were found distended with air, condensed and rubicund. The aorta, in nearly all its extent, was bright red, its parietes tumid and softened, especially within the thorax and near the diaphragm, where it was covered with varicose vessels. Its internal coat was thickened and softened. Portal, in fine, declares that there can be no doubt of the existence of aortal inflammation, and that it is always accompanied by a convulsive cough, extreme difficulty of breathing, and forcible palpitations || of the heart, terminating in sudden death.

Dr. Frank, of Vienna, assumes to himself the credit of having been the first to observe and describe the phenomena

* Dissertatio de Morbis recens Natorum Infantum Chirurgica. Lipsiæ, 1773.

† Transactions of a Society for the Improvement of Medical and Chirurgical Knowledge, Vol. I. p. 171. London, 1793.

‡ Surgical Observations, first edition, Part IV. p. 232.

§ Cours d'Anatomie Medicale, Tome III. p. 127, 128. A Paris, 1803.

|| Landré Beauvais declares palpitations to be constant accompaniments of organic lesion of the heart and large blood-vessels. Séméiotique, ou Traité des Signes des Maladies, p. 59. A Paris, 1813.

of vascular inflammation, originating from indeterminate causes. He found it in many cases of inflammatory fever, and told Corvisart,* in 1809, that he had then detected traces of aortal inflammation in nineteen subjects who had died with febrile symptoms, of the most malignant kind. He considers it to be the cause of a particular fever which has hitherto always proved fatal; and, with reference to his knowledge of this fact, he chooses to employ these assertive terms.† "*Venarum totam compagem internâ superficie undique profundè rubentes ac inflammatas nos primùm conspeximus, similesque arteriæ imprimis phlogoses partiales sub eidem circumstantiis, jam pluries ostendimus.*"

Corvisart,‡ in his pathological researches, frequently observed a redness of the inner aortal coat. This redness he describes as being more or less deep, various in its extent, but unaccompanied with any thickening of the membrane wherein it appears. He professes himself, at the same time, unable to offer any explanation of the nature and cause of this phenomenon. Mr. Hodgson, however, with his characteristic modesty, refers its development to other than an inflammatory cause. We find ourselves induced to bring his sentiments under the consideration of our readers, in his own words.

"The internal surface of arteries," says Mr. H. "often exhibits a red appearance, which does not arise from acute inflammation. The internal coat is of a deep scarlet colour, sometimes throughout the whole extent of the system; and, on other occasions, this appearance is to be observed only in circumscribed patches. It is attended with no deposition of lymph, or thickening of the vessel; and, if the internal coat be removed, the middle generally presents its natural appearance; whereas, in the cases of acute inflammation which I have examined, the middle coat has always exhibited a preternatural degree of vascularity. This red appearance of the internal surface of an artery is often observed in the vicinity of coagula, and in those instances may probably be the effect of transudation after death. But I have very frequently remarked it where no coagulum has been found in the vessel, and it is generally to be observed in arteries that have been long exposed to the air in a dissecting room. I have, however, seen it in subjects that have been inspected a very few hours after death, and cannot therefore regard it as produced merely by exposure to the air. Whether it is to be considered as a change which takes place after death, or as a morbid appearance, I am unable to determine."§

---

* Essai sur les Maladies du Cœur, p. 351.

† De Curandis Hominum Morbis.

‡ Essai sur les Maladies du Cœur et des Gros Vaisseaux, p. 350.

§ Treatise on the Diseases of Arteries and Veins, 1815, p. 7, 8.

According to Mr. Hodgson, the internal coat of an artery bears a striking analogy to serous membranes, especially in its tendency to assume the adhesive inflammation. He has occasionally met with such a state of the great blood-vessels in acute inflammations of the thoracic organs. In one case, he says, p. 6, "the aorta was, throughout, of a deep scarlet colour; the posterior mediastinum was gorged with serum, and a little above the semilunar valves, the cellular membrane that connects the coats of the aorta was distended with lymph."

We transcribe from his interesting pages a case of pneumonic inflammation, in which the disease had also affected the internal coat of the aorta, and produced an accretion of organizable lymph on its surface.

"A man," p. 5, "who had recently returned from Jamaica, where he had been severely afflicted with dysentery, was attacked with violent pneumonia, which destroyed him in the course of a few days. The cavities of the pleura were found to contain much lymph and serum. The pericardium was covered with lymph. The cells of the lungs were filled with bloody serum, and the bronchia were highly inflamed. All the thoracic viscera exhibited the effects of the highest degree of acute inflammation, which had extended also to the aorta, the inner coat of which was of a deep red colour, and a considerable effusion of lymph had taken place into its cavity. The effused lymph was very intimately connected with the internal coat of the vessel, and a plug of it had extended into the left subclavian artery, and nearly obliterated the cavity of that vessel."

Dr. Patissier,* in dissecting a subject who died of tetanus, discovered traces of inflammation in the heart and large blood-vessels, both venous and arterial. Their inner coat exhibited a bright red colour, which became paler in proportion to the distance of parts from the heart. We ourselves, in January 1820, inspected the body of an athletic young man, who was killed by the spasms of traumatic tetanus; and, in his case, we found the venous and arterial systems deeply implicated in the disease. The internal membrane of the large veins and of the anterior cardiac cavities, was purple in colour, thickened, disorganized, and easily lacerable: that of the aorta and posterior cardiac cavities, was bright scarlet, but without thickening, and possessed of its natural tenacity. We would suggest to pathologists the propriety of making the state of the vascular system, in all cases of fatal tetanus, the subject of patient and scrutinous research.

---

* Bulletin de la Faculté de Médicine de Paris, No. X. 1816.

One of the most important cases of the disease under consideration, we detailed in our third number, page 502, to which we refer. Dr. Kennedy, of Dunning, we are informed, treated four cases of arteritis complicated with visceral inflammation. We shall introduce one of these in the Doctor's own words.

" Case.—*Pathology*. A. B. a farmer, aged forty-six years, came under my care, in July, 1815, with acute pneumonia induced, he said, by exposure in a wet dress to the nocturnal air. His symptoms were urgent, and had existed, in a severe degree, nearly three days. By three venesections, performed at short intervals, he lost sixty ounces of blood, and these depletions, with the assistance of a large blister, active purging, a few liberal doses of calomel and opium, and a suitable regimen, restored him in due time to his former health.

" This man was regular in his habits, but indued with an ardent temperament, corpulent, and remarkably obnoxious to the recurrence of thoracic inflammation. Twice in 1816, he experienced an attack of the disease, and was relieved by modifications of its original treatment.

" In spring, 1817, he was with difficulty saved from falling a victim to its violence, by the employment of free depletion, vascular and alvine, together with counter-irritants, expectorants, and a diversity of the means usually administered for the purpose of rebalancing the vital actions, Ever after this attack, he was haunted by a hard cough. He experienced a constant sense of weight and fulness in his chest; his breathing was never free, and these symptoms were, at all times, aggravated by exposure to a foggy atmosphere or the air of night.

" Having incautiously reclined upon the ground, after being heated in harvest of that year, he became very ill in the evening, and past an irksome night. Next day at noon, I found him speechless, panting for breath, and exhibiting many signs of impendent apoplexy. His jugular vein being instantly opened, forty-five ounces of blood were allowed to flow before he expressed a sense of relief. This was followed by a brisk purgative, and a blister over all the chest. Being aware of an approaching exacerbation, in the evening, he requested to lose blood, and twenty ounces were abstracted from his arm. His bowels having been freely evacuated, a scruple of calomel, and two grains of opium were now administered, and his attendants enjoined to promote their operation with a full dose of castor-oil in the morning. Under such treatment, his complaint proceeded favourably till the fourth day, when he unexpectedly began to complain of a burning pain, flashing, as it were, along the internal surface of his chest. Towards evening, all his worst symptoms returned, and, at midnight, I found him afflicted by his disease in an exquisite form. He seemed exposed to instant suffocation. His face was purple, his lips livid, his eyes fixed, prominent, and vascular. All his pulses were strong, and throbbed with extreme violence. The action of his carotid and temporal arteries was visible. They beat 120 times in a minute, and their pulsations were not regular.

"Bleeding to a large extent, at this time, gave him only a transient relief. It was repeated, and unavailably assisted during the three subsequent days with every means that could be devised. The disease gathered strength as it advanced. When able to speak, the patient described the sensation in his thorax, as if it were filled with hot water. Various parts of his body were pained and disfigured by diffuse groupes of subcutaneous tubercles, which felt hard and deep-seated. Two large vesications had arisen on the calf of his right leg: they were distended with a dark sanguiniform fluid, opaque and thicker than common lymph. His vital powers sunk fast; palpitations of the heart ensued; frequent fainting followed; hiccup and convulsions supervened; and, with an agonizing struggle, but possessing his intellectual faculties unimpaired, my patient escaped from life and from suffering.

"Dissection *twenty hours after death.* Œdema nearly confined to the extremities. Another vesication similar to those observed, before death, on the right limb, was now discovered on the external aspect of the left thigh. Irregular patches of livid, disorganized textures appeared in different parts of the body and limbs; others were studded with clusters of subcutaneous tumours, various in size, firm, and round or longitudinal in form.

"*Abdomen.* With exception of slightly increased vascularity in some parts of the mesentery and duodenum, where it crosses the vertebral column, and in the peritonæal face of the diaphragm at its aortal aperture, the abdominal and pelvic organs retained no perceptible trace of disease. The spleen and pancreas, although much larger than is natural, were healthy. When incised, the vessels of the liver gave out a fluid little darker than arterial blood, but its structure seemed perfectly sound.

"*Thorax.* Several ounces of reddish-brown serum floated within the pleura, and the vessels of this membrane, especially those of its dorsal portions were finely injected. An excess of ropy sanguinolent fluid distended the pericardium, which was thickened and inflamed. The heart's external surface was overspread with a thin layer of grumous matter, resembling, in colour and consistence, an admixture of pus and blood. Its ventricular valves and membranes, particularly the posterior, exhibited many patches of vascular net-work, some of which were purple and easily ruptured. Depositions of accretive lymph had been formed in several parts of the ascending aorta, whose inner coat presented a deep crimson line, which extended over the arch and carotids, but became fainter in the subclavian artery, as far as that vessel was traced. This vascular injection, occasionally interrupted by pellicles of effused lymph, was deep and distinct over all the internal membrane of the descending aorta and its chief divergent branches, to where it gives off the inferior mesenteric artery. Beyond this, it decreased and finally disappeared at the bifurcation of the aortal trunk. Similar traces of the disease were found in the corresponding thoracic and abdominal veins. Their redness, however, was combined with a brownish tinge, and clots of a viscid wine-coloured substance adhered to some parts of their inner coat, so as to contract the diameter of their tubes. Half an inch of the thoracic duct, at its junction with the venous system, was nearly obliterated by the thickening of its texture. Traces of recent inflammation were observed on the left division of the thoracic face of the diaphragm. The left lung was condensed and hepatized; the right gorged with frothy mucus. An incohesive layer of dark orange-coloured muculent matter lined the pneumo-gastric portion of the mucous membrane, which was soft and readily torn. Some of the internal thoracic surfaces were connected with each other, by slight adhesive bands.

" *Encephalon.* Globules of a semidiaphonous fluid exuded on many parts of the dura mater, from the vessels which, in removing the calvaria, had been ruptured. Those of the pia mater were excessively injected and distinct. The arachnoid membrane was thickened and unusually resistent. Every where the meningæal vessels were turgid with blood, which issued from them when divided. Each of the ventricles contained much sanguinolent serum, and their surfaces had the velvety appearance peculiar to mucous membranes. No part of the cerebral or cerebellic organs was found in a diseased state; but, on their being incised, small beads of watery blood oozed from all parts of their structure."

We do not deem it necessary, at present, to enter into any description of the various morbid states in which arterial inflammation is prone to terminate. By inflammation of their textures, whether chronic, subacute, or acute, we regard the arterial vessels as being made liable to the deposition of organizable lymph on their internal surface; to obliteration, dilatation, disruption, and aneurysm; to a cartilaginous, and steatomatous thickening of their inner coat; to the deposition of atheromatous, calcarious, and osseous matter in any part of their structure; and to become schirrous, cancerous, fungous, ulcerated, gangrenous, or sphacelated.

Arteritis is a subject which we should regret to see neglected. It is a disease, we conceive, of no unfrequent occurrence, particularly as a complication of fever and inflammation of internal parts. If the investigation be zealously and faithfully conducted, we would almost venture to predict, that the *synochal state* will one day be regarded as symptomatic of arteritis, and the *typhoid* of phlebitis, the acute inflammation of veins.—To the latter form of disease we propose, in an early number of the Journal, to direct the attention of our readers; and, in the mean time, are willing to hope that the present sketch will not be devoid of interest or utility.

