# Errata

**Published:** 1822-03-01

**Authors:** 


					?pape 55, line 19 from bot.yor \oyos, read voffos.

				

